# 遗传性异常纤维蛋白原血症：诊治现状与挑战

**DOI:** 10.3760/cma.j.cn121090-20240326-00115

**Published:** 2024-10

**Authors:** 孜 王, 铁楠 朱

**Affiliations:** 中国医学科学院北京协和医院血液内科，北京 100730 Department of Hematology, Peking Union Medical College Hospital, Chinese Academy of Medical Sciences, Beijing 100730, China

## Abstract

遗传性异常纤维蛋白原血症（CDF）是以纤维蛋白原功能障碍为特点的最常见的一类先天性纤维蛋白原缺陷类型。由于多数患者无明显临床症状，导致其患病率被显著低估。除了出血表现外，CDF患者还可能合并有血栓事件或妊娠相关并发症。纤维蛋白原抗原测定以及分子遗传性分析有助于CDF与其他类型的遗传性纤维蛋白原缺陷症相鉴别。CDF患者的临床表现个体间差异显著，除了个人史及家族史外，目前尚缺乏有效预测其出血或血栓风险的常规实验室方法或手段，导致其临床管理困难，特别是围术期或妊娠期的CDF患者。未来需要更多的登记组研究和前瞻性研究以提高对该疾病的认识和指导患者的临床管理。

## Abstract (en)

Congenital dysfibrinogenemia（CDF）is the most common type of congenital fibrinogen disorders, characterized by dysfunctional fibrinogen. Its prevalence is significantly underestimated due to the absence of obvious clinical symptoms in most patients. In addition to bleeding manifestations, patients with CDF may experience thrombotic events or pregnancy-related complications. Fibrinogen antigen assays and molecular heritability analyses can help differentiate CDF from other types of congenital fibrinogen disorders. The clinical presentation of CDF varies significantly among individuals, and there is a lack of routine laboratory methods to effectively predict the risk of bleeding or thrombosis in these patients, in addition to their personal and family histories. This poses challenges in the clinical management of patients with CDF, particularly during the perioperative period or pregnancy. Further registry-based and prospective studies are needed to improve our understanding of this disease and guide clinical management.

遗传性纤维蛋白原缺陷症是由纤维蛋白原基因FGA、FGB或FGG基因突变导致的一大类疾病，主要包括两种类型，一类为纤维蛋白原量的减少或缺乏，包括遗传性低纤维蛋白原血症和无纤维蛋白原血症；另一类则为纤维蛋白原质的缺陷，包括遗传性异常纤维蛋白原血症和遗传性低异常纤维蛋白原血症，其中前者纤维蛋白原抗原水平正常，而活性显著下降，后者纤维蛋白原活性和抗原均有下降但活性降低更为明显。遗传性异常纤维蛋白原血症（congenital dysfibrinogenemia, CDF）是临床上最为常见的先天性纤维蛋白原缺陷症类型，由于多数患者并无临床表现，因此其患病率被显著低估。目前在国内仅少数中心开展了纤维蛋白原抗原检测，导致其诊断率进一步降低。与遗传性低纤维蛋白原血症或无纤维蛋白原血症患者不同，异常纤维蛋白原血症除了可能导致出血表现外，部分患者还可能出现血栓倾向，导致其临床处理困难。本文系统性回顾CDF的诊治现状与存在的挑战，以期对该类疾病的临床诊治提供帮助。

一、发病机制与流行病学

CDF主要为常染色体显性遗传，且多数患者为杂合错义突变的携带者。高达85％的CDF致病突变发生于FGA基因的第2外显子或FGG基因的第8外显子[1]，其中最常见的两个突变位点（热点突变）分别为FGA基因编码的第35位精氨酸（FGA Arg35）和FGG基因编码的第301位精氨酸（FGG Arg301）[2]，这两种热点突变占所有CDF患者的70％～75％，且不存在明显的种族差异[3]–[4]。既往认为遗传性纤维蛋白原缺陷症患病率较低[5]，但由于多数CDF患者无临床表现，其实际患病率可能被显著低估。近期突变数据库的资料显示，该类疾病在人群中的患病率可能高达1％。一项研究通过对基因组聚合数据库（gnomAD）中约14万名个体的外显子/基因组数据进行系统分析，确定了FGA、FGB和FGG基因的突变负担，并对遗传性纤维蛋白原缺陷的患病率进行了估算[6]。分析显示，隐性遗传性纤维蛋白原缺乏症的全球患病率可能比迄今报道的高10倍（从东亚的百万分之1到芬兰的百万分之24.5不等）。常显性遗传性纤维蛋白原缺陷的全球患病率估计为11/1000，杂合突变携带者的频率在芬兰人中为3/1000，而在非芬兰欧洲人和非洲/非洲裔美国人中为（10～20）/1000。该研究的发现表明遗传性纤维蛋白原缺陷症的真实患病率可能显著高于既往的推断。此外该研究指出杂合突变携带者虽然可能没有临床症状，但在传播遗传突变方面具有重要作用。

二、临床表现

CDF患者临床表现具有显著的异质性，约半数患者无症状因常规检查而偶然发现[3],[7]–[8]，约25％的患者有出血表现，约20％的患者发生血栓事件。出血和血栓可在同一患者并存。2015年我国一项研究纳入了102例CDF患者，其中无症状者占68.6％，出血发生率为27.5％，血栓发生率为3.9％[4]。CDF患者的出血症状一般比较轻微，致死性出血事件少见。最常见的出血症状是皮肤出血（21.47％）、月经增多（19.58％）、口腔出血（12.42％）和鼻出血（11.58％），关节出血（2.32％）、胃肠道出血（2.32％）和颅内出血（2.11％）等严重出血事件相对罕见[9]。CDF患者亦可能存在血栓风险。在一项纳入101例患者的研究中，动脉/静脉血栓的年发生率在确诊CDF后为18.7/1000且无明显性别、年龄差异，该研究观察到28例次血栓事件，其中静脉血栓20例、动脉血栓8例[3]。导致CDF患者血栓形成的可能机制包括：①异常的纤维蛋白原与凝血酶的结合能力降低，导致血液循环中游离凝血酶水平升高[10]；②异常的纤维蛋白原和组织纤溶酶原激活物或纤溶酶原的结合障碍导致纤溶受抑[11]；③纤维蛋白凝块的强度、结构和稳定性发生改变[12]。此外，替代治疗或其他合并易栓因素亦可能导致CDF患者血栓风险增加。

育龄期CDF患者发生不良妊娠及产科并发症的风险亦有增加，包括孕早期妊娠丢失、胎盘早剥、产后出血及血栓事件等。在Fibrinogest研究中CDF患者的早期流产率为13.4％，活产率76.2％，活产孕妇产科并发症发生率为13.1％，产后出血发生率为20.7％，产科并发症主要包括早产风险（5.1％）、妊娠期糖尿病（4.5％）和子痫前期（1.7％）[13]。其他多项显示，纤维蛋白原数量减少或功能下降可造成妊娠早期流产，发生率为20％～50％[3],[14]–[15]。这与纤维蛋白原在妊娠期发挥的特殊生理功能相关。纤维蛋白原/纤维蛋白可支持早期滋养细胞的增殖和扩散[16]，有利于胎盘植入[17]并维持母胎循环的发育[18]。

三、诊断与临床表型预测

（一）诊断流程

大多数的遗传性纤维蛋白原缺陷症患者是因为常规检查或术前实验室筛查发现纤维蛋白原活性（Clauss法）减低而被发现。依据纤维蛋白原活性减低程度的不同，患者还可出现活化部分凝血活酶时间（APTT）、凝血酶原时间（PT）和凝血酶时间（TT）的延长，其中以TT最为敏感。导致纤维蛋白原活性减低的主要原因包括纤维蛋白原生成减少与纤溶亢进，前者主要见于遗传性纤维蛋白原缺陷或是继发于肝病、营养不良或某些药物；后者常见于DIC、肿瘤或溶栓治疗等。通过对纤溶代谢产物如D-二聚体及纤维蛋白（原）降解产物（FDP）的测定，可首先排除原发性或继发性纤溶亢进导致的低纤维蛋白原血症。在详细询问病史及用药史排除可能导致纤维蛋白原生成不足的继发性病因后，就应考虑到患者是否存在遗传性的纤维蛋白原缺陷。患者既往出血或血栓等临床表现以及家族史有助于进一步支持遗传性纤维蛋白原缺陷症的诊断。

在诊断遗传性纤维蛋白原缺陷症后，应根据纤维蛋白原活性及抗原水平的变化进一步鉴别遗传性纤维蛋白原缺陷症的类型（表1）。通常认为当纤维蛋白原活性/抗原比值<0.7且抗原水平正常时，应考虑诊断为CDF。目前国内通常采用化学发光免疫分析法进行纤维蛋白原抗原检测。由于纤维蛋白原抗原检测并未得到广泛开展，对于无条件进行抗原测定的医院亦可考虑采用PT衍生法进行CDF的筛查。Miesbach等[19]研究发现PT衍生法测得的纤维蛋白原数值与免疫法测到的纤维蛋白原抗原数值呈正相关性。Xiang等[20]–[21]评估了PT衍生法/Clauss法纤维蛋白原比值用于筛查异常纤维蛋白原血症的效力，认为PT衍生法/Clauss法纤维蛋白原比值大于1.43诊断遗传性异常纤维蛋白原血症的敏感性和特异性可达100％。

**表1:** 各类纤维蛋白原缺陷症的实验室诊断[25]

类型	APTT	PT	Fbg功能（Clauss法）	Fbg功能（PT衍算法）	Fbg抗原
无纤维蛋白原血症	显著延长	显著延长	测不出	测不出	测不出
低纤维蛋白原血症	正常或延长	正常或延长	降低	通常降低	降低
异常纤维蛋白原血症	正常或延长	正常或延长	通常降低	通常正常	正常
低异常纤维蛋白原血症	正常或延长	正常或延长	降低	通常降低	降低

**注** APTT：活化部分凝血活酶时间；PT：凝血酶原时间；Fbg：纤维蛋白原

通过家系成员测定或基因检测可进一步确定遗传性纤维蛋白原缺陷症以及进一步的分型诊断。对FGA、FGB、FGG基因进行基因测序，如发现基因变异，可检索人类纤维蛋白原数据库（GFHT，https://site.geht.org/base-fibrinogene/）[22]和相关综述[23]–[24]明确是否已有报告以及相应的纤维蛋白原缺陷类型。

（二）基因型是否可预测临床表型

在确定CDF的诊断后，如能准确预测每位患者的临床表型，将有助于制定个体化的疾病管理策略。目前已知8个CDF相关基因变异与血栓风险明确相关（表2）：Caracas V、Ijmuiden、Bordeaux、New York I、Melun、Nijmegen、Naples（纯合突变时）和Dusart[10],[26]。根据ISTH先天性纤维蛋白原缺陷诊断和分类指南[26]，携带该类突变的患者被归为3B亚型（血栓相关异常纤维蛋白原血症），因其强烈的血栓倾向而被视为遗传性易栓症而非出血性疾病。另一方面，有一些研究发现了与出血相关的突变类型[27]。然而，在其他一些相同突变案例中，患者并未表现出明显的出血症状。这表明CFD止血失衡的机制可能受到多种因素的影响而不是仅由患者基因型决定。Li等[9]系统性挖掘了文献和突变数据库，并对出血相关的基因型与可能出血机制进行了概述。然而遗憾的是，在接近80％的CDF患者中存在的热点突变（FGA Arg35和FGG Arg301）并不能够提示患者未来的出血或血栓风险。因此，在大多数CDF患者中基因型检测结果并不能有效预测临床表型，也不能据此来指导患者的临床管理。

**表2:** 与异常纤维蛋白原血症血栓形成强烈相关的纤维蛋白原突变类型[1],[26],[28]

命名	基因	外显子	c.DNA	新生蛋白
Bordeaux	FGA	5	c.1390C>T	p.Arg458Cys
Caracas V	FGA	5	c.1595C>G	p.Ser551Cys
Dusart	FGA	5	c.1717C>G	p.Arg573Cys
Ijmuiden	FGB	2	c.130C>T	p.Arg44Cys
New York I	FGB	2	第2外显子缺失	p.Del39-102
Nijmegen	FGB	2	c.220C>T	p.Arg74Cys
Naples	FGB	2	c.292G>A	p.Ala98Thr
Melun	FGG	8	c.1169A>T	p.Asp390Val

**注** Naples仅纯合突变与血栓相关

（三）实验室检查是否可预测临床表型

功能性（如测量纤维蛋白肽裂解速率、纤维蛋白原体外聚合和溶解特性）和结构性（如扫描电子显微镜、激光扫描共聚焦显微镜、渗透性、黏弹性特性、单个纤维的力学特性和原子力显微镜）实验室检查有可能为患者临床表型的预测提供一些间接依据[1]。例如，根据恒定压降和流速下的凝块渗透性可估算纤维蛋白纤维网络的孔隙大小，应用扫描电子显微镜可评估纤维直径，进而提示血凝块的脆性（出血或高凝）[25]。整体止血功能检测方法，如血栓弹力图通过全血的黏弹性变化可整体评估血凝块的功能和结构，提供有关纤维蛋白网特性的动态值，亦可应用来监测纤维蛋白原替代的疗效[4]。需要注意的是，血栓弹力图对CDF患者纤维蛋白网结构和功能的变化的敏感性有限，具体效能还需要扩大化的研究来验证。在不久的将来，一些新技术例如飞行时间质谱（TOFMS）也可能有助于CDF患者的诊断与管理[29]。

四、CDF的管理与治疗

因为CDF的诊断率低，目前尚缺乏对于CDF治疗的前瞻性临床研究数据，治疗指南证据级别多为专家共识。CDF患者的临床症状个体间差异显著，常规实验室检查结果并不能有效预测患者的出血或血栓风险，因此治疗方案主要应基于患者既往的临床表现和家族史进行个体化制定。

（一）日常生活管理

主要包括对于出血及血栓两方面事件的管理。对于既往无症状且无阳性出血家族史患者，不推荐常规接受纤维蛋白原的预防性替代治疗。轻微出血事件通常首选局部压迫或缝合止血，必要时可考虑辅助止血药物治疗如抗纤溶药物[1]。月经增多的患者亦可考虑性激素治疗。血栓方面，如患者携带任何表1中血栓相关突变或存在一级亲属血栓家族史（ISTH分型3B），则视为血栓高危人群，在高危情况下应考虑预防性抗凝[30]，而其他CDF患者通常仅建议机械性预防。CDF患者发生血栓时的抗凝方案仍存在争议，低分子肝素为主要推荐药物[1]，亦有多项个案报道提供了应用直接口服抗凝药的经验[31]–[32]。由于患者常常存在基线PT延长，故不建议应用华法林抗凝。通常建议抗凝周期为3～6个月，如为ISTH分类3B亚型，建议进一步延长抗凝时间或终身抗凝[21]。

（二）急性出血事件的处理

CDF患者发生急性大出血事件时，建议立即进行纤维蛋白原替代治疗，可选择新鲜冰冻血浆、冷沉淀或冻干人纤维蛋白原浓缩物，其中冻干人纤维蛋白原提升纤维蛋白原水平的效率最高，同时可避免感染及输液容量过大，应作为替代治疗的首选[33]。替代治疗在处理CDF相关出血时面临的主要挑战是如何确立可量化的指标以指导治疗。目前仍是借鉴低/无纤维蛋白原血症的指南，将目标纤维蛋白原活性定为1.0～1.5 g/L[30],[34]–[35]。可选择的经验性输注方案包括人纤维蛋白原20～50 mg/kg，或冷沉淀15～20 ml/kg，或新鲜冰冻血浆15～30 ml/kg[21]，基于人体纤维蛋白原半衰期为3～5 d，建议每周输注2次以维持纤维蛋白原水平。输注的人纤维蛋白原仍可与CDF患者体内的异常纤维蛋白原发生相互作用，输注效果有可能低于无/低纤维蛋白原血症患者。Chandler等[36]报道了一种新的校正算法，基于患者TT纠正试验结果计算出校正参数并根据该校正参数调整纤维蛋白原浓缩物的给药剂量。该给药方案的可行性仍需要得到更大规模的前瞻性研究进一步验证。

（三）围手术期管理

对于拟行手术或有创操作的CDF患者，术前是否需要预防性输注以及替代治疗的目标目前仍存有争议。事实上除了止血功能外，纤维蛋白原在手术后伤口愈合中也有非常重要的作用[37]。我们建议综合患者及直系亲属的既往出血表现、手术相关出血风险及纤维蛋白原活性水平等因素来制定个体化的治疗策略。对于出血风险较大的手术（颅脑、胸腹、截肢、关节置换等）或既往有出血表现的CDF患者，建议将术前纤维蛋白原活性峰值提升至1.0～1.5 g/L以上，术后维持治疗方案可以根据手术恢复情况及出血量等酌情调整。对于拟行小手术特别是既往无明显出血表现的患者，通常不需要进行术前预防性替代治疗，必要时可考虑抗纤溶药的辅助治疗。由于部分CDF患者具有血栓风险，因此应警惕过度替代治疗导致围术期血栓风险增加[30]。

（四）妊娠期管理

女性CDF患者妊娠期的管理比围术期管理更为复杂，除了可能的出血和血栓风险外，妊娠期的不良事件还包括习惯性流产、死产、胎盘早剥、宫内生长受限等。对于计划妊娠的CDF患者，应向其充分交代妊娠期可能的并发症以及遗传风险，并与患者充分沟通妊娠期可能的管理方案。一项针对遗传性纤维蛋白原缺陷症合并妊娠患者的文献综述显示，30.8％的患者在妊娠期接受过纤维蛋白原输注，预防性、治疗性输注分别占81％、19％[14]。目前多数学者不建议对无症状患者常规进行纤维蛋白原替代治疗，而是推荐定期监测患者的纤维蛋白原活性及抗原水平和胚胎发育情况[21]。如患者妊娠期发生出血事件，可考虑给予纤维蛋白原替代治疗，维持纤维蛋白原活性水平大于1.0～1.5 g/L。对于既往有复发性妊娠丢失的CDF患者，或是在妊娠期间出现胎盘功能不全时，在无其他可能病因解释的情况下，可与患者充分沟通纤维蛋白原替代治疗的选择。分娩环节是该类患者妊娠期最富挑战的时刻，最佳分娩方式尚无定论。因CDF多为常染色体显性遗传，胎儿有50％的概率携带突变基因，应警惕困难分娩时负压吸引与产钳可能增加胎儿颅内出血的风险。麻醉方式方面，未经替代治疗不建议行椎管内麻醉，但目前尚无该类患者椎管内麻醉导致出血的报道。通常建议分娩前纤维蛋白原活性应提升至1.5 g/L以上。CDF患者在分娩后，尤其要警惕产后出血和血栓事件的风险。发生产后出血的患者，可给予纤维蛋白原浓缩物替代和抗纤溶治疗。所有CDF患者分娩后均应考虑常规给予抗深静脉血栓的机械预防。对于既往有血栓史的患者，应考虑整个妊娠期及分娩后的药物预防。多学科团队联合管理和诊治对于CDF患者顺利度过妊娠期至关重要。

综上所述，由于多数CDF患者无明显临床症状而导致就诊率低，其真实患病率仍然未知。对于该类患者的诊断，除了应排除获得性纤维蛋白原缺陷外，主要应与遗传性低纤维蛋白原血症相鉴别。CDF患者的临床表现具有显著的异质性，除了个人史及家族史外，目前尚缺乏有效预测其出血或血栓风险的常规实验室方法或手段，导致其临床管理困难，特别是围术期或妊娠期的CDF患者。目前亟需大规模的登记组研究和前瞻性研究，以提供高质量循证医学证据指导和优化该疾病的临床诊疗。
